# Supplementary material for: Identification and functional analysis of non-coding regulatory small RNA FenSr3 in Bacillus amyloliquefaciens LPB-18
Source: PeerJ. 2023 May 15;11:e15236. doi: 10.7717/peerj.15236 (PMC10194069; doi:10.7717/peerj.15236)
Supplement: Supplemental Information 4 [file peerj-11-15236-s004.zip › KO/CK-vs-T1_map/map04215.html]

KEGG PATHWAY: Apoptosis - multiple species - Reference pathway


|  |  |
| --- | --- |
| **Apoptosis - multiple species - Reference pathway** |  |

[
Pathway menu
| Organism menu
| Pathway entry
| Show description
| User data mapping
]

Apoptosis is an evolutionarily conserved process used by multicellular organisms to developmentally regulate cell number or to eliminate cells that are potentially detrimental to the organism. The major players are caspases, caspase inhibitors, members of the Bcl-2 family of pro- and anti-apoptotic proteins and adaptors of the Ced-4/APAF-1 type. Mammals, by comparison with Caenorhabditis and Drosophila, exhibit highly complex extrinsic and intrinsic pathways for apoptosis induction. However, recent analyses of whole genome sequences from cnidarians (e.g. Hydra) suggest that the caspase and Bcl-2 families were already highly complex in cnidarians and that Caenorhabditis and Drosophila lost many of the genes involved in apoptosis.

|  |  |  |
| --- | --- | --- |
| Reference pathway | 184% 150% 122% 100% 82% 67% 55% | 图片下载 |
